# Sensory Cues Modulate Smooth Pursuit and Active Sensing Movements

**DOI:** 10.3389/fnbeh.2019.00059

**Published:** 2019-04-08

**Authors:** Ismail Uyanik, Sarah A. Stamper, Noah J. Cowan, Eric S. Fortune

**Affiliations:** ^1^Department of Mechanical Engineering, Johns Hopkins University, Baltimore, MD, United States; ^2^Department of Biological Sciences, New Jersey Institute of Technology, Newark, NJ, United States

**Keywords:** active sensing, weakly electric fish, electrosensation, sensory cue, sensorimotor system, smooth pursuit

## Abstract

Animals routinely use autogenous movement to regulate the information encoded by their sensory systems. Weakly electric fish use fore–aft movements to regulate visual and electrosensory feedback as they maintain position within a moving refuge. During refuge tracking, fish produce two categories of movements: smooth pursuit that is approximately linear in its relation to the movement of the refuge and ancillary active sensing movements that are nonlinear. We identified four categories of nonlinear movements which we termed scanning, wiggle, drift, and reset. To examine the relations between sensory cues and production of both linear smooth pursuit and nonlinear active sensing movements, we altered visual and electrosensory cues for refuge tracking and measured the fore–aft movements of the fish. Specifically, we altered the length and structure of the refuge and performed experiments with light and in complete darkness. Linear measures of tracking performance were better for shorter refuges (less than a body length) than longer ones (>1.5 body lengths). The magnitude of nonlinear active sensing movements was strongly modulated by light cues but also increased as a function of both longer refuge length and decreased features. Specifically, fish shifted swimming movements from smooth pursuit to scanning when tracking in dark conditions. Finally, fish appear to use nonlinear movements as an alternate tracking strategy in longer refuges: the fish may use more drifts and resets to avoid exiting the ends of the refuge.

## 1. Introduction

Animals often use movement to control and modulate the sensory information they receive. These movements alter an individual's position relative to sources of sensory stimuli in the environment and generate autogenous feedback (Han et al., 2009; Maimon et al., 2010). Such movements are a form of “active sensing” in which animals expend energy for the purpose of sensing (Nelson and MacIver, 2006). A hallmark of active sensing behavior is that it is modulated in relation to available sensory information (Hille et al., 2001; Gao et al., 2003; Visalberghi and Néel, 2003; Raburn et al., 2011). For example, *Eigenmannia virescens*, a species of weakly electric fish, dramatically alters its refuge tracking behavior in relation to the availability of sensory cues.

**Figure 1:**
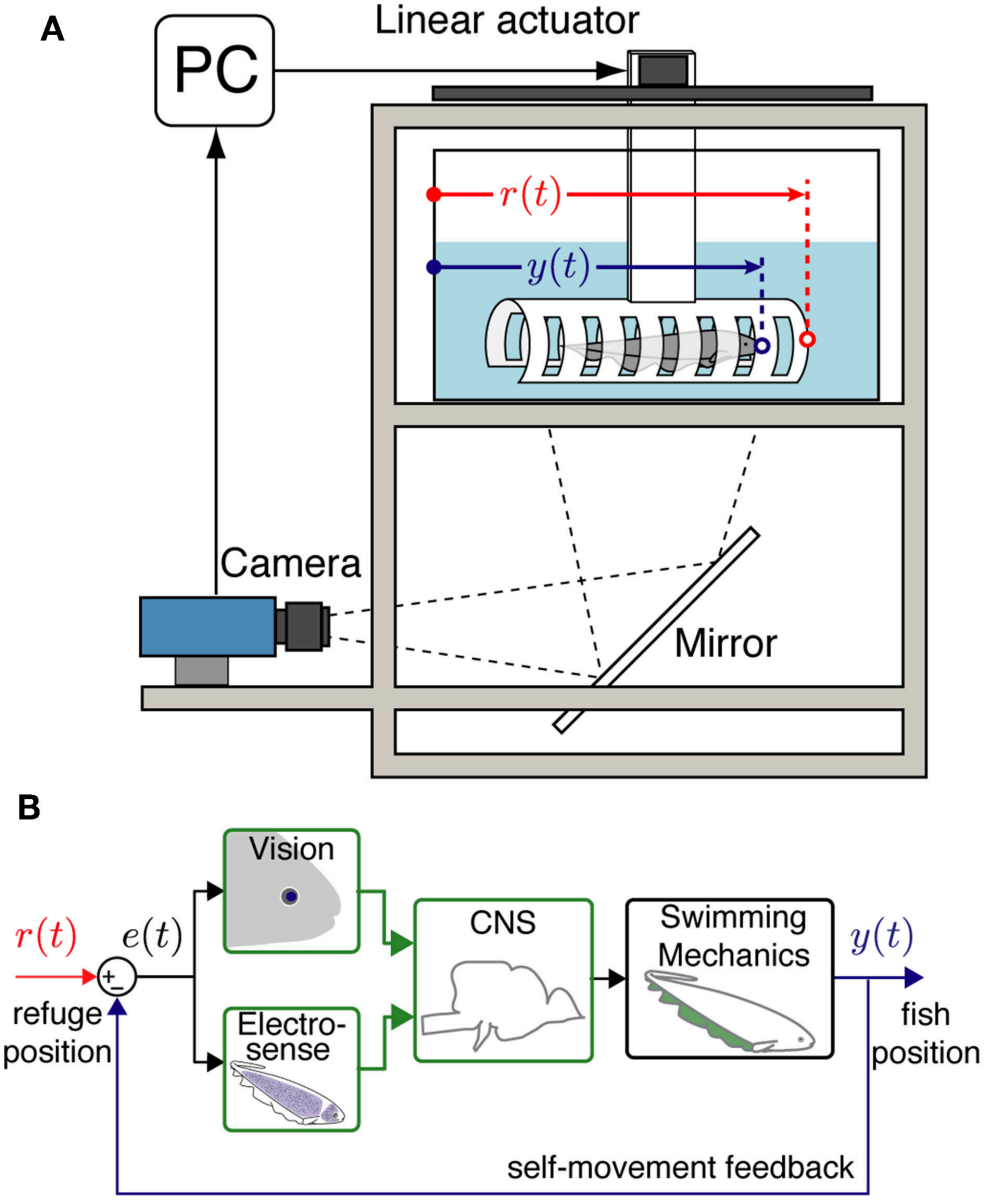
Experimental setup and system model. **(A)** An illustration of the data collection system with an *Eigenmannia virescens* swimming inside a moving refuge. The refuge movements are controlled by a PC through a stepper motor. A camera is positioned to record the fish and refuge movements from below via a mirror. *r*(*t*) is the position of the refuge and *y*(*t*) is the position of the fish. **(B)** A block diagram schematic of the closed-loop refuge tracking behavior. *e*(*t*) is the difference between *r*(*t*) and *y*(*t*) that can be detected by the visual and electrosensory systems. Information from the fish's sensory systems are transformed by neural circuits in the central nervous system (CNS) to control the swimming mechanics of the fish. Movements of the fish y(t) produce autogenous feedback based on the resulting sensory slip, *e*(*t*) = *r*(*t*) − *y*(*t*).

In refuge tracking behavior, fish track the position of a longitudinally moving refuge by swimming forwards and backwards to stay within it (see Figure 1). This behavior has been observed both in natural habitats, as fish hide within the trunks of fallen palm trees and other vegetative litter, and in the laboratory, as fish maintain their position within PVC tubes and other refuges provided for them. Fish tune their relative reliance on visual and electrosensory cues during tracking depending on the relative salience of the two cues (Sutton et al., 2016), but can rely on electrosensory cues when tracking in complete darkness (Stamper et al., 2012).

Fish can generate smooth linear tracking movements but they also generate a class of fore–aft movements that are not linearly related to the movement of the refuge. These movements generate relative displacement between the refuge and the fish, termed “sensory slip,” which can be percieved by its electrosensory (Pedraja et al., 2018) and visual systems. Sensory slip has previously been termed “sensory error” under the presumption that any relative movement between the fish and refuge was due to limitations in the ability of the fish to track the refuge perfectly (Cowan et al., 2014). However, recent evidence demonstrates that fish actively maintain non-zero sensory slip via these fore–aft movements (Biswas et al., 2018). In sum, refuge tracking behavior comprises two complementary components, smooth pursuit tracking, which ideally seeks to minimize sensory slip, and active sensing, which seeks to generate ongoing sensory slip to attain the information needed to remain with the refuge.

The sensory feedback that electric fish perceive as they move through their environment depends on the physical structure of nearby objects (Babineau et al., 2007; Hofmann et al., 2014; Dangelmayer et al., 2016; Gottwald et al., 2017; Schumacher et al., 2017). Because the receptive fields of electrosensory receptors are spatially confined (Krahe and Maler, 2014), the lengths and spatial heterogeneity of the walls of refuges can have dramatic impacts on information encoded by each receptor and by downstream neural populations in the brain (Hofmann and Chacron, 2018). Electroreceptors can potentially encode such spatial structure, which the fish could use to determine its position within the refuge. For example, the presence of holes (“windows”) in the walls of the refuge result in spatial variance of the strength of the electric field within the refuge. Such variance can modulate the activity of electroreceptors in relation to the relative movement of the fish and refuge, potentially enhancing the fish's ability to detect sensory slip.

How do fish modulate their movements for tracking in relation to the availability of sensory cues? Theoretically, fish could optimize the movements they use to track refuges with different lengths and degrees of spatial heterogeneity via a variety of strategies. For example, fish could increase the magnitudes of active sensing movements as electrosensory cues are reduced in longer and less spatially heterogeneous refuges. Alternatively, fish could adopt a number of different possible tracking strategies, such as tracking the ends of the refuge where the changes in the electric field are likely to be strongest, or remaining within the refuge by making rapid forward or backward corrections when the end of refuge is detected by the tail or head, respectively, to avoid leaving the refuge.

To examine the linear and nonlinear strategies that fish use to track the position of a moving refuge, we measured the movements of *Eigenmannia* in relation to the length of the refuge, presence or absence of windows in the walls of the refuge, and presence or absence of visual cues.

## 2. Materials and Methods

Adult *Eigenmannia virescens* (10 − 15 cm in length) were purchased from commercial vendors and housed according to published guidelines (Hitschfeld et al., 2009). The water temperature in the housing and test tanks were maintained at ~ 27 °C with a conductivity range of 150 − 300 μ*S*/ cm. All experimental procedures were approved by the Johns Hopkins and Rutgers animal care and use committees and followed guidelines established by the National Research Council and the Society for Neuroscience.

For experiments, individual fish were transferred to a test tank. This tank was equipped with a computer-controlled linear stepper motor to move the refuge and a video camera to record the fish movements (see Figure 1A). Fish were allowed to acclimate to the test tank for 2 − 24 h prior to experimental trials. During trials, if the fish left the refuge and did not return within 1 min, the overhead lights were turned on and the fish was gently guided back into the refuge using an aquarium net (Rose and Canfield, 1993). Subsequently animals often returned to the refuge when the overhead light was turned on.

### 2.1. Experimental Apparatus

The experimental setup (Figure 1A) was similar to that used in previous reports (Cowan and Fortune, 2007; Roth et al., 2011; Stamper et al., 2012). Eight different refuges were used for the experiments. Refuges were machined from 2” × 2” gray rectangular PVC tube at four different lengths; 7.5, 12.5, 15, and 22.5 cm. We had two refuges at each of these lengths. Refuges with “windows” had rectangular holes, 0.625 cm width, 2.0 cm spacing machined into each side. Windows provide additional visual and electrosensory cues than refuges with solid sides, “no windows.”

The bottom faces of the refuges were removed to allow video recording from below. Video was captured at 30frames·*s*^−1^ with 1280 × 1024 resolution using a high-speed camera (pco.1200s, Cooke Corp, Romulus, MI) with a Micro-Nikkor 60*mm* f/2.8D lens (Nikon Inc., Melville, NY) and Camware software (Cooke Corp, Romulus, MI). For each trial, the refuge was moved back and forth according to predefined sinusoidal trajectories by a linear stepper motor (IntelliDrives, Inc, Philadelphia, PA) driven by a Stepnet motor controller (Copley Controls, Canton, MA). The actuator trajectories and camera triggering were synchronized using a Multifunction DAQ (USB-6221, National Instruments, Austin, TX) and controlled with custom Matlab scripts (MathWorks, Natick, MA).

### 2.2. Experimental Procedures

Individual fish (*N* = 4) were tested with a series of refuge movement trajectories. Trajectories were sinusoidal at frequencies of 0.01, 0.55, and 1.15 Hz with a mean velocity amplitude of 1.2*cm*·*s*^−1^. Each trial was 60 s in duration with an initial 10*s* ramp up and final 10*s* ramp down periods. Data from the ramp periods were excluded from analysis.

Each fish experienced trials under two illumination conditions, light or dark. “Light” trials refer to use of white light (300 − 500 lux) in addition infrared light and “dark” trials refer to use of only infrared light. The pairing order for illumination and refuge frequency was randomized. Each fish completed 3 − 5 trials for each of the following conditions:

- illumination: light or dark
- refuge frequency: 0.01, 0.55 or 1.15 Hz
- refuge structure: with windows or without windows
- refuge length: 7.5, 12.5, 15, or 22.5 cm.

We performed a total of 710 trials. The data for each fish was collected over 1 − 2 weeks. Inter-trial intervals were at least 2 min. The sequences of refuge trajectories, lighting, and refuge structures were randomized. As we have observed in previous studies, fish did not show long-term adaptation or changes in tracking performance over time (Cowan and Fortune, 2007; Roth et al., 2011; Stamper et al., 2012). That said, these fish have been shown to rapidly (on the order of seconds) adapt to the statistics of refuge movements (Roth et al., 2011). In the present study, we only used predictable, single-sine stimuli (Roth et al., 2011).

### 2.3. Data Analysis

Fish and refuge position were digitized using custom tracking code implemented in Matlab. For each trial, we measured the trajectory of the refuge, *r*(*t*) and the fish, *y*(*t*). Fish position was measured using a custom template-based video tracking algorithm centered on a “ventral white spot” found between the pectoral fins, just caudal to or below the gills of *Eigenmannia*.

The Discrete Fourier Transform (DFT) represents the time-domain signals *r*(*t*) and *y*(*t*) as complex-valued functions of frequency, *R*[ω] and *Y*[ω]. These complex numbers can also be represented in polar coordinates in terms of their magnitude, |*Y*[ω]|, and phase ∠*Y*[ω]. For the single sine wave input trajectories, the DFT *R*[ω] is represented as a discrete spike at the refuge frequency and zero at all other frequencies. In contrast, the DFT of the fish movement *Y*[ω], typically as power over a broader range of frequencies (0.1 to 2.0 Hz; e.g., see Results, **Figure 6**).

Frequency-response plots describe the response of a system by comparing the output signal, *Y*[ω], to the input signal, *R*[ω], using two measures, gain and phase. For each frequency ω_0_, the gain is calculated as the ratio of the signal magnitudes, |*Y*[ω_0_]|/|*R*[ω_0_]|, and phase is computed as the difference between signal phases, ∠*Y*[ω_0_] − ∠*R*[ω_0_]. The frequency-response plot is only evaluated at the stimulus frequency, as the gain ratio and phase lag are not defined where the stimulus magnitude is zero, i.e., *R*(ω) = 0.

We further analyzed the refuge and fish movements by dividing them into “epochs.” The epoch boundaries were determined based on zero-crossing of the velocity signals, where positional movement changes direction. For each of these epochs (*n* = 31269) we calculated a three dimensional vector of duration, amplitude, and mean velocity for further analysis. Note that duration and amplitude already constrain the velocity: velocity data were included for visualization purposes. To identify linear smooth-pursuit epochs, we compared this 3D vector to the expected duration, amplitude, and mean based on the refuge trajectory using outlier analysis (see published Matlab code for details). Once the smooth-pursuit epochs were removed, we used K-means clustering to classify the remaining nonlinear epochs.

We then hand checked the classification of each epoch as follows. First, we made time-domain plots of each fish trajectory in which each epoch was displayed using colors that identified its category. Each epoch was reclassified as needed using a custom Matlab user interface. For example, some high velocity events that immediately followed drift events were initially classified as “scanning” via K-means clustering and we reclassified such events as “reset.” Likewise, small oscillations at the peaks and troughs of otherwise smooth pursuit were reclassified as ‘wiggle’ epoches. Because each epoch has been validated by human inspection, this data is appropriate for use as a training set for future use with machine learning algorithms.

## 3. Results

**Figure 2:**
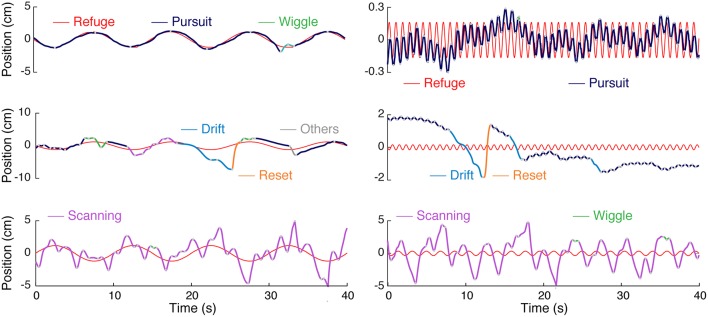
Categories of movements produced by *Eigenmannia virescens* during refuge tracking behavior. Smooth pursuit or simply pursuit (blue) have an approximately linear relation to refuge movement (red). The other categories—wiggle (green), drift (cyan), reset (orange), and scanning (magenta)—are nonlinearly related to the stimulus trajectory. The category “other” (gray) was used as a catch-all for a variety of infrequent, relatively low-amplitude, movements. The types of movements are illustrated for both low (left) and high (right) frequency refuge trajectories.

When tracking the position of a moving refuge, *Eigenmannia* produce smooth-pursuit swimming movements that are roughly linearly related to the movement of the refuge and nonlinear ancillary active sensing movements (Figure 2). We identified four categories of nonlinear movements: wiggles, scanning, drift, and reset (see section 3.1). We measured the effects of light, refuge length, and presence/absence of windows in the refuge walls on linear tracking performance (see section 3.2), tracking position (see section 3.3), and nonlinear behaviors (see section 3.4).

### 3.1. Categories of Movements for Refuge Tracking

The movements that fish made while tracking were divided into uni-directional “epochs” and categorized in a two-step process. In the first step, a simple automated clustering algorithm was used followed by visual inspection and hand reclassification of segments. Figure 2 illustrates the five stereotyped movements we identified in *Eigenmannia* in both low and high frequency cases; smooth pursuit or pursuit, scanning, wiggle, drift, and reset. Smooth-pursuit epochs can be defined as the movements where the fish motion follows the moment-to-moment velocity of the refuge. The tracking gain, the ratio of fish's amplitude to the refuge amplitude, varied in relation to the frequency of the movement, as described in previous reports (Cowan and Fortune, 2007; Stamper et al., 2012).

Nonlinear movements were segregated into four categories. High amplitude back and forth movements are labeled “scanning.” Scanning is the most common form of nonlinear movements, and are the movements that have been highlighted in previous work (Stamper et al., 2012; Jun et al., 2016; Biswas et al., 2018). In experiments in which the refuge was moved at low frequencies (see Figure 2), scanning movements were imposed on the low frequency tracking movements performed by the fish. In contrast, in experiments with higher-frequency refuge movements (Figure 2), the scanning movements are imposed on what appears to be the mean position of the refuge over the trial.

The second category of movements, labeled “wiggle,” are characterized by small-amplitudes and high frequencies. Wiggles are produced mostly at the peaks (lowest velocities) of the fish's sinusoidal tracking movements resulting in short-distance movements less than the body length of the fish. The third category of movements, “drift,” are low-frequency, high amplitude movements. The majority of drift movements are made in the backwards (toward the tail) direction and commonly have durations of more than 1 s. The fourth category of movements, “reset,” are commonly seen immediately following drift movements. These are high-velocity forward (toward the head) movements that are typically produced when the fish was moving beyond the end of the refuge. The distances traveled during scans are on the order of the refuge length, whereas distances traveled drifts and resets are smaller than the refuge length in general. Finally, the fish occasionally produce idiosyncratic movements that do not easily fit into one of these four categories, which we have labeled “others” for completeness.

**Figure 3:**
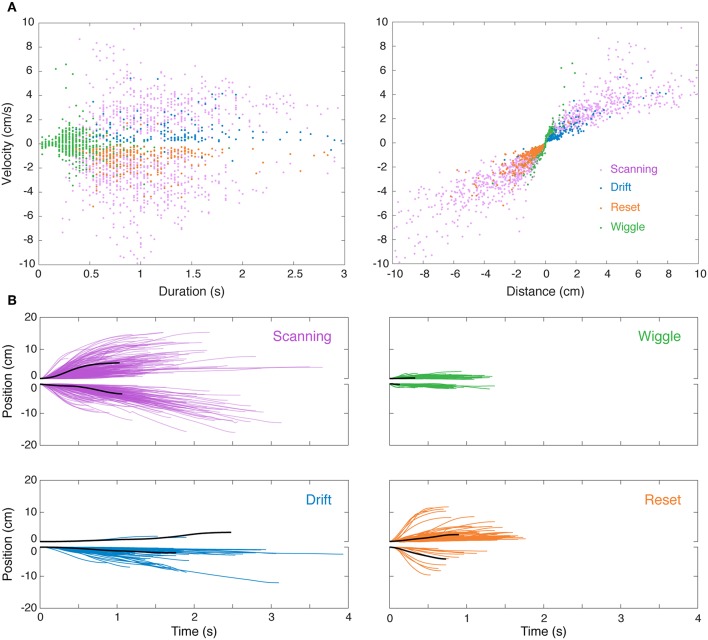
Visualization of nonlinear movements in feature space and time domain. **(A)** After separately identifying and removing linear “smooth pursuit” epochs, the remaining nonlinear epochs were clustered in the 3D feature space of duration, velocity, and distance. This is visualized in two 2D projections (velocity–duration, *left*; velocity–distance, *right*), excluding “others” for clarity. **(B)** Sample single-epoch trajectories of four categories of nonlinear movements. 3000 randomly selected epochs of the 31269 epochs recorded in this experiment are shown. The solid black lines are the epoch closest to the centroid (in 3D feature space) of the cluster. Forward (positive position values) and backward (negative position values) movements are illustrated separately for clarity. Colors match the movement types in **(A)**.

Figure 3A shows time vs. distance for the four main nonlinear movement types. We observed differences in nonlinear movements in relation to the direction of movement. Forward scans were produced with higher velocities than backward scans. Drift and reset movements were preferentially produced in the reverse and forward directions, respectively.

We observed significant variability in trajectories both within and between clusters. Indeed, the clusters, which were initially segmented using a K-means algorithm based on measures of duration, position, and velocity, are overlapping (see Figure 3B) and are more accurately described as a continuum. The distinction between many of these movements lies in the context in which they were produced, e.g., while in the middle of the refuge or at the edge, which was determined during manual reclassification of tracking epochs.

### 3.2. Refuge Tracking Performance

**Figure 4:**
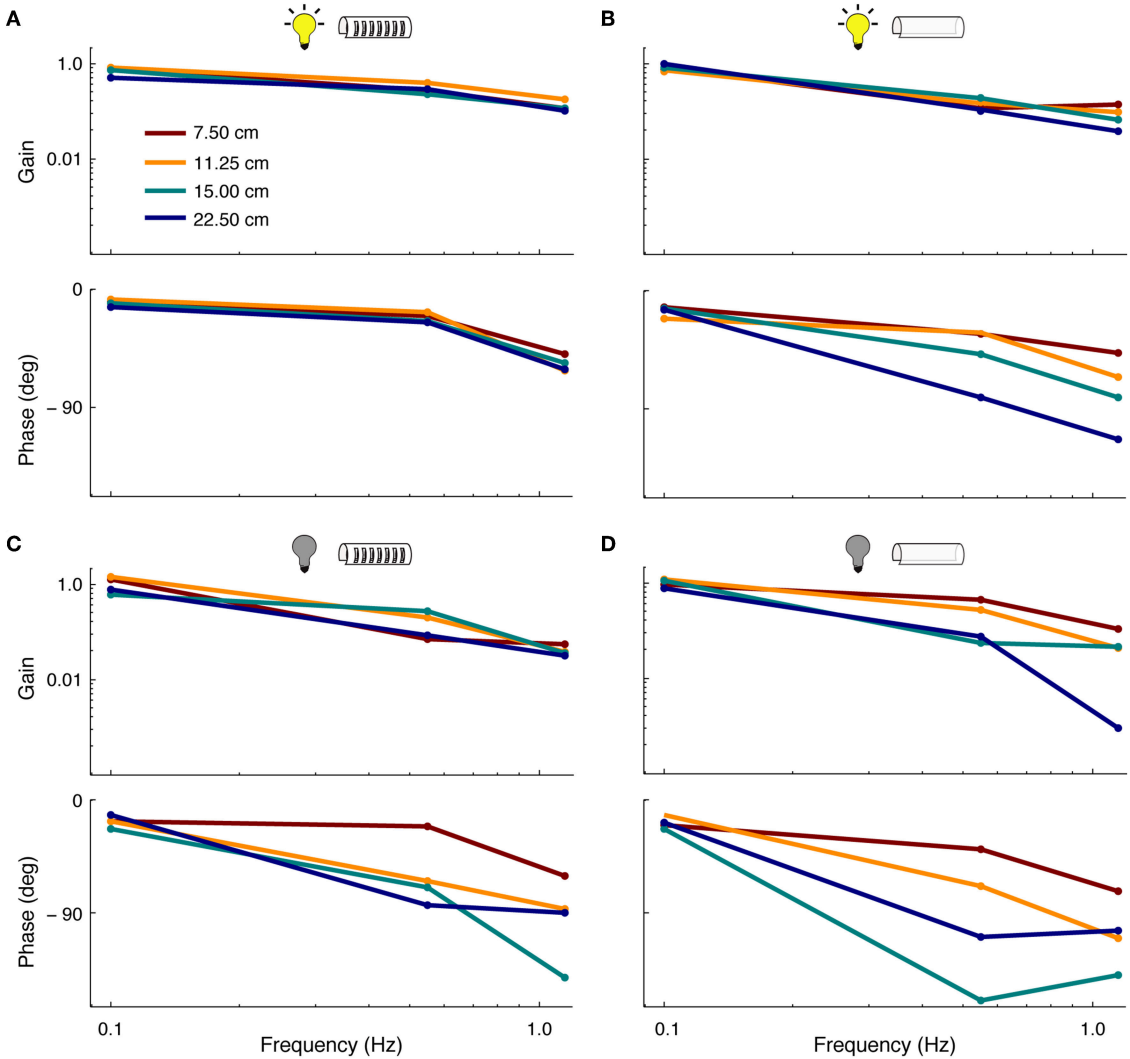
Effect of sensory cues on smooth-pursuit performance. Frequency response functions showing the gain (top) and phase (bottom) of fish during smooth pursuit. Colors correspond to the four lengths of the refuge. **(A)** Tracking performance in light conditions in refuges with windows. **(B)** Tracking performance in light conditions in refuges without windows. **(C)** Same as in **(A)** but in complete darkness.**(D)** Same as in **(B)** but in complete darkness.

Linear tracking—i.e., smooth pursuit—performance was assessed in relation to lighting conditions and features of the refuge (Figure 4). In general, linear tracking performance was found to be similar to previous reports (Cowan and Fortune, 2007; Roth et al., 2011; Stamper et al., 2012)—fish had higher frequency-response gains and smaller phase lags when tracking in the light vs. dark. The length of refuges with windows had little effect on tracking gain and phase in the presence of visual cues. However, the removal of windows from the refuge led to increases in tracking phase lags, especially at higher stimulus frequencies (see Figure 4B). In the longest refuge without windows, phase lags were observed at up to 120°.

In the absence of visual cues, we observed more variability in tracking response across different refuge lengths. In dark, the length of refuges with windows affects both the tracking phase and gain. This effect is increased for refuges without windows (see Figure 4D). For example, at 0.55 Hz, the tracking gain was reduced from ~ 0.9 to 0.3 as the refuge length was increased.

### 3.3. Refuge Length Affects Fish Position

**Figure 5:**
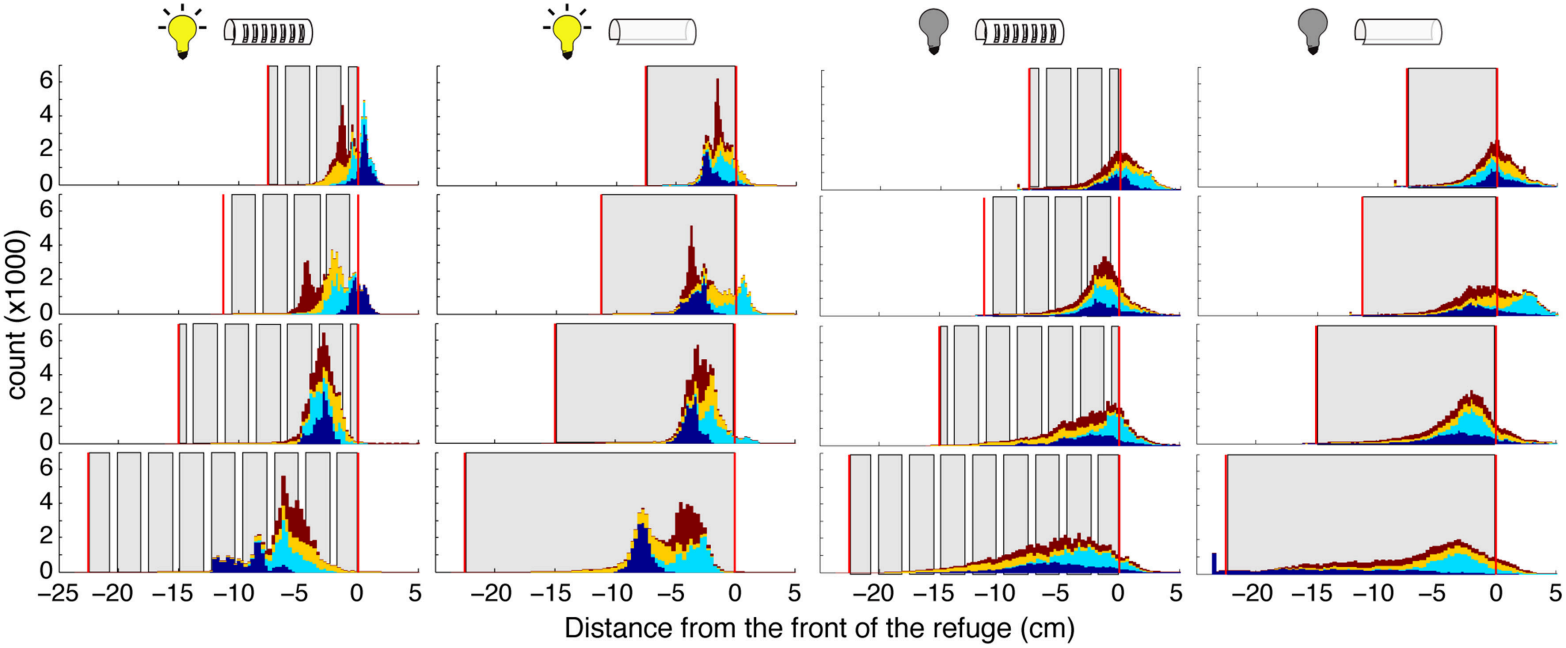
Distribution of fish position within the refuge. Histograms showing the position of the fish position. (specifically the “ventral white spot”; see section 2) Each color corresponds to a different fish. Histograms are stacked; no data are occluded. Gray areas represent the walls, and vertical red lines the ends, of the refuges. Experimental conditions (light and dark, with and without windows) are indicated by the icons above each column. The “front” of the refuge, as determined by the direction of the fish, is set as 0 so that positive movements are toward the head and front of the refuge, and negative movements are toward the tail and end of the refuge.

We measured the position of fish within the refuge during tracking experiments. Figure 5 illustrates the position of each of the four fish that were tested. In general, each of the fish placed their head near one end of the refuge, even when refuges were less than the body length. Fish maintained head position closer to the end of the refuge and poked their heads outside of the refuge more often for shorter refuges than for longer.

Illumination had a major effect on position within refuge during tracking. In the dark, fish position was more evenly spread throughout the refuge, and fish poked their head outside of the refuge more frequently. These effects are likely a result of the production of nonlinear movements in the dark. We do not see an effect of illumination on the fish's mean location within the refuge. Further, we did not observe an effect of the presence of absence of windows on the fish's location preference.

### 3.4. Sensory Cues Shape Active Sensing Movements

**Figure 6:**
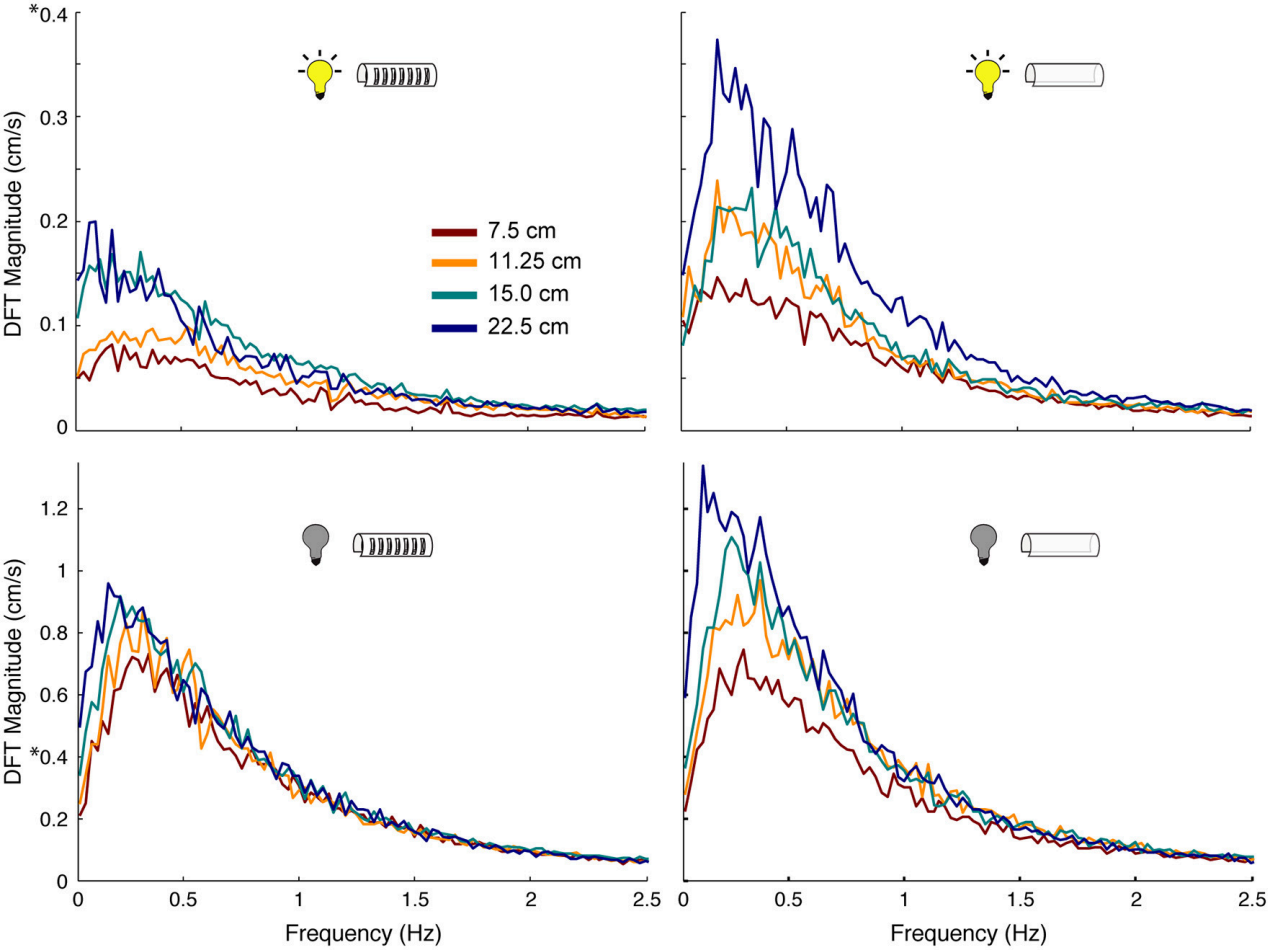
Effect of sensory cues on magnitude of nonlinear movements of the fish. Power spectra of tracking movements in which the peak at the stimulus frequency has been removed, thereby leaving only the power associated with the nonlinear components of the behavior. Colors correspond to different length refuges. Experimental conditions are indicated by the icons in each plot. Note the scale difference between light (DFT Magnitude to 0.4 cm/s) and dark conditions (DFT Magnitude to 1.3 cm/s): fish made more nonlinear movements in the dark than in the light, highlighted by 8 on the y-axis.

The production of active sensing movements increased when fish tracked in the dark when compared to in the light. Using Fourier analyses, we found that fish performed 3–5 times more active sensing movements while tracking in the dark compared to in the light (Figure 6). Similarly, increases in length of the refuge, especially for refuges without windows, caused an increase in the magnitudes of active sensing movements of the fish. The peak power of these nonlinear movements was consistent across all experimental conditions, and was between 0.1 and 0.4 Hz.

**Figure 7:**
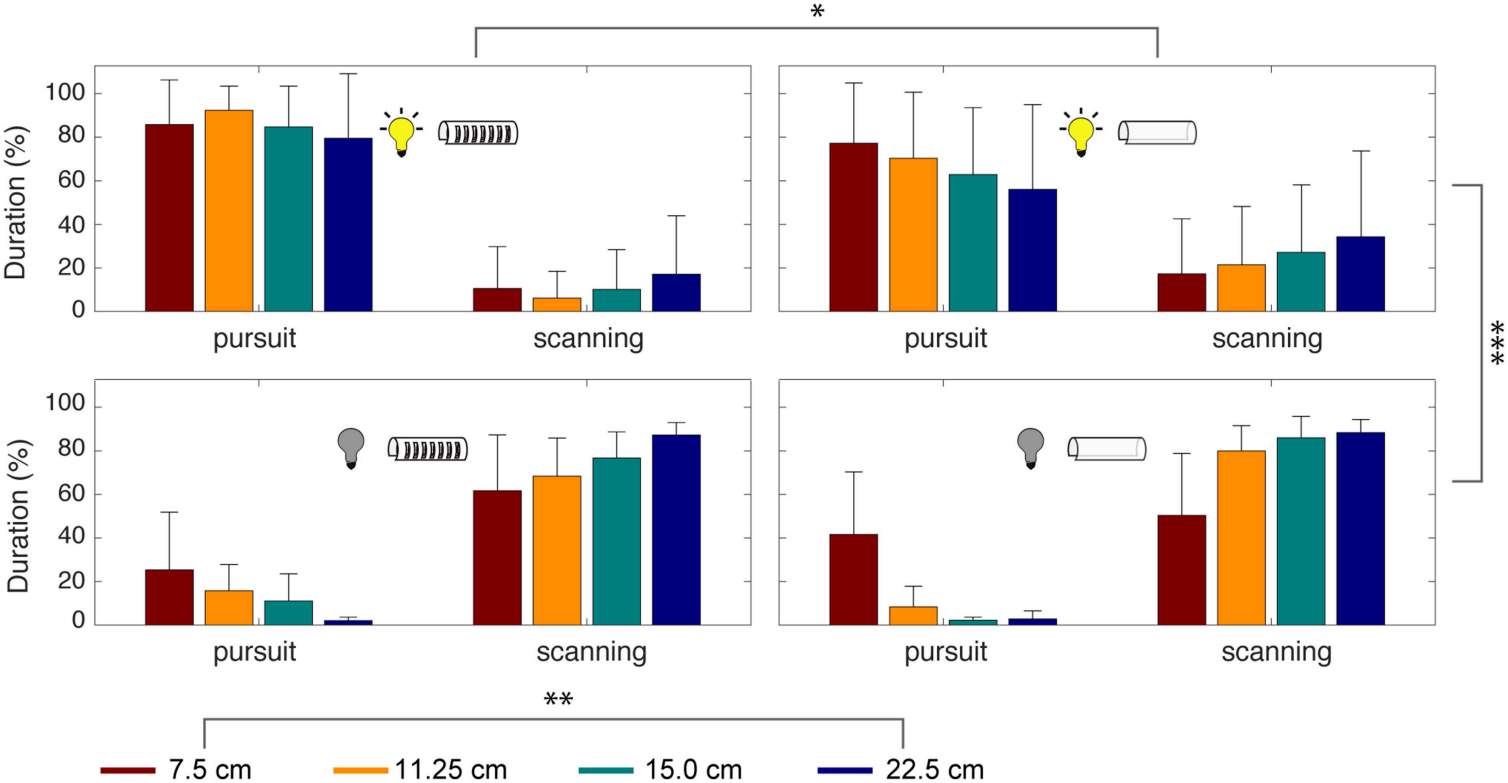
Time spent in smooth pursuit and scanning depends on sensory cues. Percentage of time spent during smooth pursuit vs. scanning movements. Experimental conditions are indicated by the icons and refuge length by color. Error bars indicate standard deviation. A four-way ANOVA revealed significant effects of illumination (^***^p < 0.0005), refuge length (^**^p < 0.005), and presence or absence of windows (^*^p < 0.05). For the low-frequencies tested, the frequency of refuge movement did not have a significant effect.

We also measured the durations of tracking movements across experimental conditions. Smooth pursuit and scanning were produced significantly more than the other three categories of movements, accounting for 91.3% of the time spent during refuge tracking. To evaluate the effect of the experimental parameters on smooth pursuit and scanning, we conducted a four-way ANOVA analysis considering illumination, presence/absence of windows, refuge lengths, and refuge movement frequency (Figure 7).

This analysis revealed that the most important parameter for tracking was illumination [*F*_(1,144)_ = 263, *p* = 2.92 × 10^−29^] followed by refuge length [*F*_(3,144)_ = 6.01, *p* = 8.45 × 10^−4^]. The presence of windows had a significant effect on tracking performance [*F*_(1,144)_ = 5.08, *p* = 0.0264] but refuge movement frequency did not [*F*_(2,144)_ = 0.37, *p* = 0.6923]. Finally, the combination of illumination and windows also contributes significantly [*F*_(1,144)_ = 5.72, *p* = 0.0187]. In other words, there is little effect of windows on smooth pursuit in the light, but in the dark we see a significant effect of windows on smooth pursuit.

**Figure 8:**
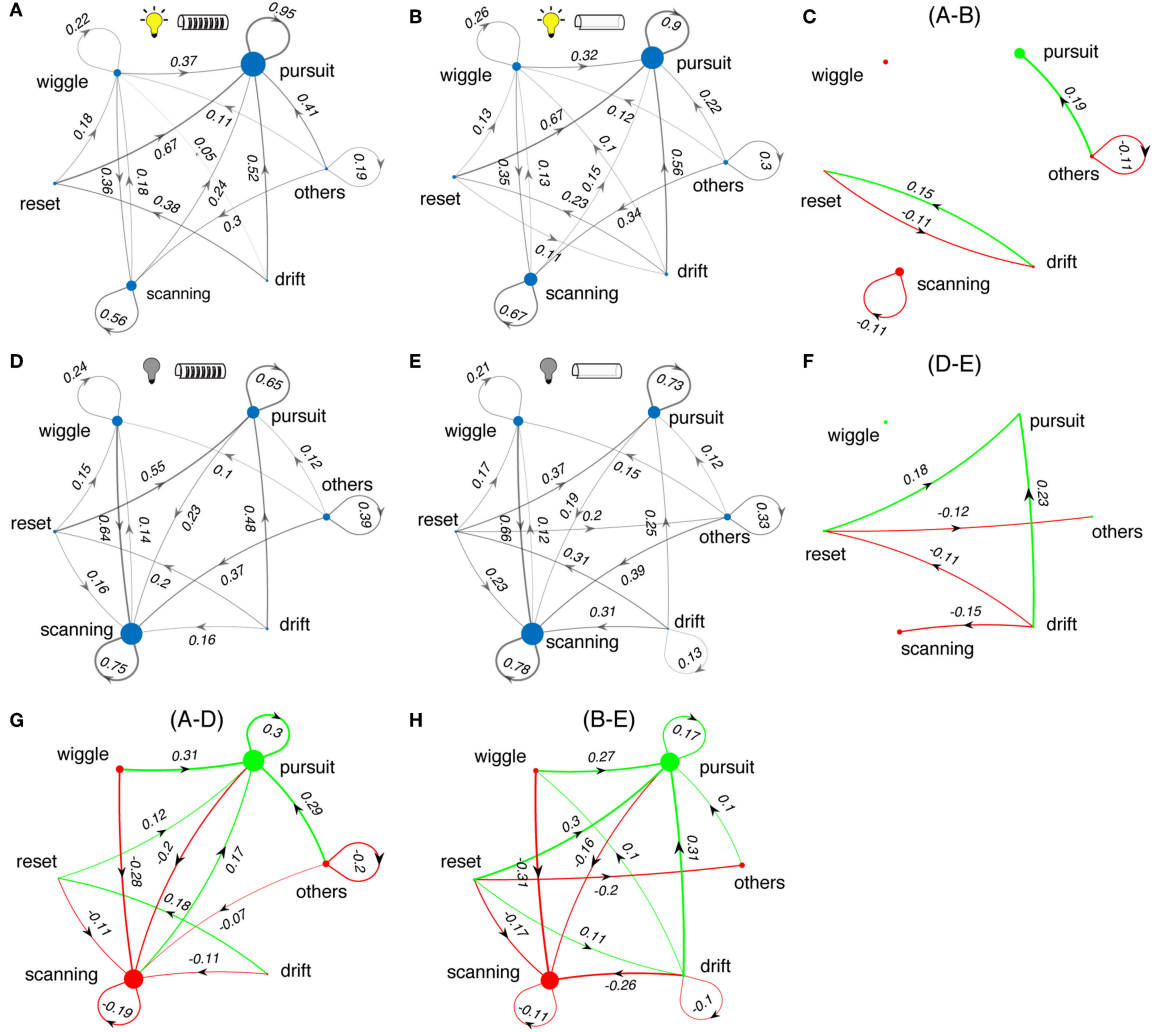
Changes in sequences of tracking behaviors. **(A,B,D,E)** State transition diagrams between the different movement types of *Eigenmannia* under various sensory conditions. Experimental conditions are shown as icons for panels. Note that these data include all length refuges. Difference plots illustrate the effects of changes in the lightning conditions **(C,F)** and presence and absence of windows **(G,H)** on state transition graphs. Green and red colors correspond to increasing and decreasing effects, respectively. Sizes of dots and thickness of lines are proportional to the number of occurrences of each epoch type or transition, respectively.

Next we examined the effects of sensory cues on the sequencing of the categories of tracking movements. We calculated the transition probabilities between movement types across different test conditions. Figure 8 shows state transition graphs for light and dark conditions, and with and without windows. The area of each node represents the percentage number of occurrences for each movement type, while the lines between nodes represent the transition probability from the source node to target node. The lines with probability <10% are removed for clarity. The green and red colored plots (right and bottom, Figures 8C,F,G,H) illustrate the changes in tracking behavior between experimental conditions.

In light conditions, fish produced sequences of smooth-pursuit movements as reflected by self-transition probabilities of 0.90 or greater (Figures 8A,B). The presence or absence of windows to refuges had little effect on tracking behavior (Figure 8C). In dark conditions, fish produced sequences of scanning movements and sequences of smooth-pursuit movements, with self-transition probabilities of between 0.65 and 0.78 (Figures 8D,E). The presence of windows had a significant effect in the dark—transitions from drifts and resets to smooth pursuit occurred far more often in the presence of windows (Figure 8F).

**Figure 9:**
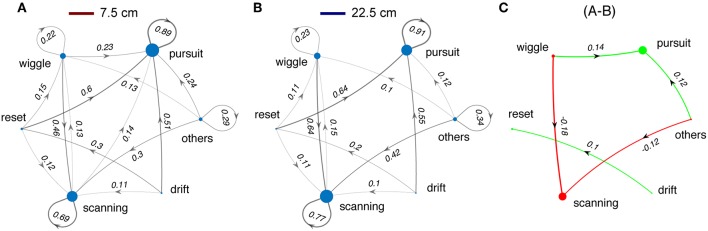
Changes in sequences of tracking behaviors in relation to refuge length. Same format as Figure 8. **(A)** Data for all trials with refuge length of 7.5 cm. **(B)** Data for all trials with refuge length of 22.5 cm. **(C)** The small differences between **(A,B)** justify collapsing across refuge length for Figure 8.

Switching from light to dark had profound effects on tracking behavior. Fish shift from making smooth-pursuit movements in the light to scanning movements in the dark. There are also significant changes in the transition from wiggles, resets, and drifts—in the dark fish transition more to scans and in the light to smooth pursuit. Shorter refuges (Figure 9) lead to increased smooth pursuit, especially in the dark.

## 4. Discussion

A general feature of active sensing is that it is modulated in relation to both the categories and spatiotemporal features of sensory information available to the animal (Hille et al., 2001; Gao et al., 2003; Visalberghi and Néel, 2003; Raburn et al., 2011). In this study, we characterized the relations between variations in sensory information available to *Eigenmannia* and the categories of active sensing movements they produce. These findings may provide insights into the mechanisms for feedback control of behavior in animals.

*Eigenmannia* track the longitudinal movement of a refuge using at least 5 categories of swimming movements. The first category of movement is known as smooth pursuit which is characterized by its similarity to the movements of the refuge. Like smooth pursuit in visual systems (Lisberger et al., 1987), fish's movements have a generally linear relationship to the stimulus—the fish's movements are at the same frequencies as the stimulus but with changes in gain and phase. In smooth pursuit, the electrosensory image is stabilized on the electroreceptors embedded in the skin.

The four other categories are not linearly related to the stimulus frequency. As described previously, fish produced significantly more of these nonlinear movements when tracking in the dark compared to the light (Stamper et al., 2012). Scanning movements are most similar to previously described movements for active sensing. Wiggle movements appear to be smaller amplitude scans that are performed as fish velocities approach zero. Wiggles may also be a form of active sensing that helps maintain sensory slip (Biswas et al., 2018). Drift and reset movements may also be used for active sensing, but may also contribute to an alternate strategy for tracking the refuge in which the fish, rather than stabilizing the electrosensory image on its receptors, uses large movements to avoid departures from the refuge.

### 4.1. Measures of Linear and Nonlinear Performance

Tracking behavior includes both linear and nonlinear components, requiring a combination of analytic approaches. Frequency domain analyses capture the linear performance of the fish. These analyses effectively ignore nonlinear components of tracking as the movement of the fish is assessed only in relation to stimulation frequencies. Nevertheless, the continuous production of nonlinear movements by fish can potentially affect linear measures, most commonly in relation to phase. For example, linear analysis shows that fish had longer phase lags in dark for long, windowless refuges, which is likely related to the increased production of nonlinear movements in these experimental conditions.

Frequency domain analyses do not capture other salient features of fish tracking behavior. For instance, information about the position of the fish within the refuge is lost during frequency-response estimation. The position of the fish in the refuge may be important because it is possible that fish may adopt different strategies for tracking that could affect these sorts of linear analyses. Consider, for example, that the movements of the refuge used in this and previous experiments were always zero-mean (no translation of the refuge). As a result, the most efficient strategy in terms of distance traveled is to remain still. Such a strategy would require, at a minimum, that the fish learn the statistics of the refuge movement, which is known to occur (Roth et al., 2011). However, given that *Eigenmannia* continuously produce counter-propagating waves in its ventral ribbon fin, there may be little additional energetic cost for making small movements within the refuge.

In previous experiments (Cowan and Fortune, 2007), we assumed that fish actively track the position of the refuge. However, fish could use an alternative strategy in which they avoid exiting the refuge. In this strategy, fish do not track the moment-to-moment position of the refuge, but rather make compensatory movements when the fish finds itself at the edges or outside of the refuge. Here we found evidence for both of these strategies. Fish routinely perform linear tracking of the refuge, but also performed drift and reset movements that appear to be behaviors that are used to avoid departure from the refuge.

### 4.2. Sensory Context

We found that sensory cues had a significant impact on the linear tracking, e.g., smooth pursuit, and nonlinear movements, e.g., active sensing. Visual cues had the greatest impact, similar to previous findings (Stamper et al., 2012). When visual cues were present, animals preferentially performed smooth pursuit. In the absence of visual cues, fish increased the production of active sensing movements. Indeed, our analysis revealed that absence or presence of light works as a behavioral switch from smooth pursuit to active sensing.

The production of active sensing movements in trials without visual cues was affected by features of the refuge that likely impact electroreception. We observed significant changes in tracking behavior in relation to the presence or absence of windows when the fish tracked in the dark but not in the light.

What are the functional roles of each of the categories of movements? Scanning and wiggle movements are often produced continuously, and may be used for the generation and maintenance of sensory slip (Biswas et al., 2018). Scans may be used as a form of local search to identify an “optimal” location within the refuge for tracking. Drift and reset movements may be used to avoid the ends of the refuge, although their relative infrequency suggests this is not a dominant strategy in refuge tracking behavior. Future analyses could incorporate models of electrosensory image formation (Babineau et al., 2007) with models of sensory afferents (Chacron et al., 2003) to identify and characterize the impacts of each category of active sensing movements on sensory encoding (Nelson and Maciver, 1999).

### 4.3. Observability

Illumination, length of the refuge, and availability of windows each had significant effects on the smooth pursuit performance of fish. We believe that these factors effect the “observability” of the movement of the refuge. In control theory, observability is a metric to evaluate how well the internal states of a system can be inferred using measures of output signals from the system (Doyle et al., 2013). Our feedback control model for the refuge tracking behavior (Figure 1B) considers the difference between the refuge and fish position *e*(*t*) as the input to the central nervous system: observability of *e*(*t*) is critical for the control of tracking responses. This information is perceived by their visual and electrosensory systems.

High-pass filtering by sensory receptors can lead to perceptual fading for slowly moving stimuli, thereby reducing observability. To avoid this problem, animals routinely use ancillary active sensing movements (Lederman and Klatzky, 1987; König and Luksch, 1998; Peters et al., 1999; Madsen et al., 2004; Najemnik and Geisler, 2005; Ghose and Moss, 2006). A mathematical transfer function model of *Eigenmannia* also showed that such movements can help the fish to recover observability (Kunapareddy and Cowan, 2018). Similarly, the addition of salient features within a refuge, such as windows, may also improve observability of the sensory slip *e*(*t*). In this experiment, long refuges without windows were expected to provide fewer sensory cues and thereby have reduced observability. Our results do not directly support this hypothesis—refuge length had a greater impact than the presence or absence of windows on tracking performance.

Details of our experimental approach may have reduced our ability to detect the effects of windows and refuge length on observability. First, in refuges both with and without windows, the edges of the refuges always provided distinct, high-contrast sensory cues which may have provided sufficient observability. Second, we used sinusoidal movements of the refuge at single frequencies—a predictable stimulus. Because the stimulus was predictable, the fish may have had reduced reliance on sensory feedback, thereby reducing the apparent role of observability in tracking behavior (Roth et al., 2011). Future experiments could employ stimuli with more complex, less predictable trajectories. Finally, we used a symmetric pattern for the refuge windows. It is possible that the uniform window spacing results in less distinctive sensory cues for tracking. Future experiments could examine the contributions of spatial heterogeneity on recovery of observability by using refuges windows that vary in width and spacing.

## Data Availability

An archived version of the datasets and analysis code supporting this article will be made available through the Johns Hopkins University Data Archive with the following doi: 10.7281/T1/C6MCOX (https://archive.data.jhu.edu).

## Author Contributions

SS: data collection; IU and SS: data analysis, curation, writing original draft and visualization; NC and EF: supervision and funding acquisition; All authors conceptualization, writing review and editing.

### Conflict of Interest Statement

The authors declare that the research was conducted in the absence of any commercial or financial relationships that could be construed as a potential conflict of interest.
